# Self-reported symptoms after COVID-19 vaccination. Distinct sex, age, and geographical outcomes in Lebanese and Italian cohorts

**DOI:** 10.1007/s11739-023-03321-9

**Published:** 2023-06-15

**Authors:** Mohamad Khalil, Leonilde Bonfrate, Agostino Di Ciaula, Piero Portincasa, The LEITvax Group^, Hala Abdallah, Michela Capurso, Ilaria Galerati, Soukayna Hayek, Hala Khalifeh, Edoardo Mastandrea, Antonino Noto

**Affiliations:** Clinica Medica “A. Murri” & Division Internal Medicine, Department of Precision and Regenerative Medicine and Ionian Area (DiMePre-J), University “Aldo Moro”, Policlinico Hospital, Piazza G. Cesare 11, 70124 Bari, Italy

**Keywords:** COVID-19, Vaccines, Side effects, Sex, Lebanon, Italy

## Abstract

Following the COVID-19 discovery in December 2019, different vaccines were authorized in 2021 in Italy and Lebanon, but side effects and the impact of sex and age remained partly explored. We designed a web-based “Google Form” questionnaire to record self-reported systemic and local side effects up to 7 days after 1st and 2nd dose of the vaccine in two distinct Italian and Lebanese cohorts. Twenty-one questions in Italian and Arabic languages explored the prevalence and severity of 13 symptoms. Results were compared with respect to living country, timing, sex, and age classes. A total of 1,975 Italian subjects (age 42.9 ± SD16.8 years; 64.5% females) and 822 Lebanese subjects (age 32.5 ± SD15.9 years; 48.8% females) joined the study. The most common symptoms in both groups were injection site pain, weakness, and headache after the 1st and 2nd doses. The rate of post-vaccinal symptoms and the severity score were significantly higher in females than in males and progressively decreased with increasing age following both doses. We find that among two populations from the Mediterranean basin, the anti-COVID-19 vaccine generates mild age and sex-dependent adverse effects, with ethnic differences and prevalent symptoms rate and severity in females.

**Supplementary Information:**

The online version contains supplementary material available at 10.1007/s11739-023-03321-9.

## Introduction

The COVID-19 pandemic caused by the coronavirus SARS-CoV-2 became a real challenge to the public health system worldwide since the year 2019 [1], spreading to more than 200 countries worldwide [2–6].

Coronaviruses are enveloped RNA viruses broadly distributed among humans, other mammals, and birds, which can cause respiratory, enteric, hepatic, and neurologic diseases [7]. Based on the difference in protein sequences, Coronaviruses are classified into four genera (alpha-CoV, beta-CoV, gamma-CoV, and delta-CoV), among which the beta-CoV genera contains most human coronaviruses (HCoVs), and is subdivided into four lineages (A, B, C and D) [8, 9]. There are seven HCoVs including SARS-CoV-2 and they have a zoonotic origin from bats, mice, or domestic animals [8, 10]. Despite the importance of all HCoVs, SARS-CoV-2 is becoming of leading relevance. Since its appearance, the pandemic has caused high mortality rates and economic losses all over the world [11].

Until now, no vaccines have been shown to be highly effective against infection with any beta-CoV, the family that includes SARS-CoV-2 [12]. Thus, the success of potential vaccines to prevent disease and to limit further spread of infection is a key player in tempering the pandemic outbreak. Researchers developed various types of candidate vaccines with the release of the genetic sequence of the SARS-CoV-2 virus. These include inactivated viral vaccines, protein subunit vaccines, mRNA vaccines, and recombinant viral vector vaccines [13].

During the COVID-19 pandemic, many of COVID-19 vaccines were developed and underwent pre-clinical and clinical trials. However, public interest in mRNA vaccines has arisen greatly, and the mRNA-based vaccine Comirnaty from Pfizer and BioNTech was the first vaccine receiving emergency authorization by the U.S. Food and Drug Administration (FDA) on December 11, 2020, and by the European Medicines Agency (EMA) on December 21, 2020 [14].

Worldwide, the use of COVID-19 vaccines was initially authorized for emergency use and the side effects were not clearly recorded. Common side effects were expected but specific side effects linked with COVID-19 vaccines remained uncertain. In this context, survey studies are needed for a comprehensive evaluation of the relationships between vaccine administration, related undesired effects, vaccine safety, and distinct geographical, age-dependent, and sex responses.

This study represents the first survey following early COVID-19 vaccines administration in two distinct geographic areas, comparatively considering possible differences due to sex and age.

## Subjects and methods

A tailored, anonymous, web-based questionnaire (see below) was used to conduct a survey in two different target populations free-living in Italy or in Lebanon. The survey was conducted between March and July 2021 distributing the questionnaire by social-media platforms (WhatsApp, Email, Facebook) or by face-to-face interview. Participation was on a voluntary basis and following random sampling. Included in the survey were subjects older than 18 years without a previous SARS-CoV-2 infection, who received at least one dose of COVID-19 vaccine.

The two groups consisted of 1975 Italian and 822 Lebanese individuals, within the framework of ongoing scientific and clinical collaborations. The protocol was approved by the local Ethics Committee, University of Bari ‘Aldo Moro’ (study number 6752, protocol number 0031044), and by ClinicalTrials.gov (NCT05735769).

## Questionnaire

A specific questionnaire (Supplementary Material Table S1) was prepared in Italian and translated into Arabic (by M.K.). The survey questionnaire was designed using “Google Form” and the link was shared by social-media platforms (WhatsApp, Email, Facebook) or during face-to-face interview with people without web access.

The questionnaire consisted of 21 items exploring demographic data, details about timing of COVID-19 vaccine, and onset of 13 possible symptoms within 7 days after administration of the 1st and 2nd dose of vaccine. Symptoms included: allergic-urticarial reaction, anaphylaxis, diarrhea (i.e., more than 3 bowel habits a day), fever (i.e., body temperature > 37 °C), headache, insomnia, irritability/nervousness, pain at the injection site (with or without redness, swelling and induration), lymphadenopathy, myalgias, skin rash, vomiting, and weakness.

Besides symptoms, the following aspects were also evaluated: use of antipyretic/analgesic drugs, recourse to a doctor, admission to emergency room without or with subsequent hospitalization.

The individual intensity of three most frequent symptoms, i.e., headache, pain at the injection site, and myalgias was assessed semi-quantitatively by a modified Visual Analogue Scale (VAS) included in the question: “How would you rank the intensity of your symptom (symptom) from 0 = no to 100 = max”. A cumulative severity score was also calculated by the sum of the intensity (VAS) of each of the three symptoms (headache, pain at the injection site, and myalgias) and a score attributed to antipyretic/analgesic drugs, recourse to a doctor, admission to emergency room without or with subsequent hospitalization. The final cumulative severity score was therefore obtained by the following formula:

∑(VAS headache (0–100) + VAS pain at the injection site (0–100) + VAS myalgias (0–100)) + antipyretic use (10 point) + other analgesic use (10 point) + recourse to a doctor (10 point) + admission to emergency room without hospitalization (10 point) + hospitalization (10 point).

The score ranged from 0 to 350 and was calculated after the 1^st^ and the 2^nd^ dose.

## Statistical analysis

Data are expressed as mean and standard deviation (SD) for continuous variables, or as proportions and percentages for categorical variables. The chi‐square test (proportions), and the Mann–Whitney U test were employed to evaluate intra‐ or inter‐group differences, as appropriate. All statistical analyses were performed using NCSS software (NCSS 2021, LLC. Kaysville, Utah, USA, ncss.com/software/ncss). Statistical significance was declared if a two‐sided P‐value was < 0.05 [15].

## Results

A total of 2797 subjects returned the questionnaires, i.e., 1975 Italian subjects and 822 Lebanese subjects. All received questionnaires were completely filled. Among the enrolled subjects, 1758/1975 (89.0%) Italians and 722/822 (87.8%) Lebanese had received both doses. Detailed information on enrolled subjects divided according to living area, vaccine dose, age, and prevalence/N. of symptoms is depicted in Table 1.

**Table 1:** Characteristics of the 2797 participants according to country, vaccine doses, and sex distribution

	All	Males	Females
Italian cohort			
1st dose N	1975	702 (35.5%)*	1273 (64.5%)*
Age (yrs)	42.9 ± 16.8^a^	45.2 ± 17.0*	41.7 ± 16.5*
Symptomatic N (%)	1483 (75.1%)^a^	479 (68.2%)*	1004 (78.9%)*
N. of symptoms	1.9 ± 1.7^a^	1.7 ± 1.4*	2.1 ± 1.8*
2nd dose	1758/1975 (89.0%)	631 (35.9%)*	1127 (64.1%)*
Age (yrs)	43.2 ± 17.1	45.6 ± 17.0*	41.8 ± 17.0*
Symptomatic N (%)	1403 (79.8%)^a^	441 (69.9%)*	926 (82.2%)*
N. of symptoms	2.9 ± 2.0	2.5 ± 1.9*	3.2 ± 2.1*
Lebanese cohort			
1st dose N (%)	822	421 (51.2%)	401 (48.8%)
Age (yrs)	32.5 ± 15.9^a^	33.9 ± 16.4*	31.0 ± 15.2*
Symptomatic N (%)	307 (37.3%)^a^	121 (28.7%)*	186 (46.4%)*
N of symptoms	5.0 ± 3.9^a^	4.6 ± 3.9	5.2 ± 3.9
2nd dose	722/822 (87.8%)	354 (49.0%)	368 (51.0%)
Age (yrs)	31.2 ± 14.7	32.3 ± 15.1	30.2 ± 14.2
Symptomatic N (%)	431 (59.7%)^a^	172 (48.6%)*	259 (70.4%)*
N of symptoms	5.1 ± 3.8	4.5 ± 3.9*	5.4 ± 3.7*

Data are expressed as numbers (N), mean ± SD, percentages (%)

Asterisks indicate significant differences between sexes (*) p < 0.01; symbol (^a^) indicates significant differences between countries after same dose p ≤ 0.01. Statistics by Mann–Whitney test (means) or by chi‐square test (proportions)

Regarding the Italian participants, 1387 (70%) were health workers (surgeons or veterinarians, nurses, hospital stuff, and psychologists), 240 (12%) were school staff (students, teachers), and the remaining 348 (18%) had other jobs.

The Lebanese cohort was composed by 297 (36%) health workers, i.e., doctors, surgeons, nurses, and hospital staff, 179 (22%) workers of the Lebanese red cross, 167 (20%) school staff (students and teachers), and 179 (22%) with other occupations.

The type of vaccines and the prevalence of symptoms in subjects divided according to the type of received vaccines are reported in Supplementary Table S2. Regarding the 1st dose, in both cohorts the majority of subjects received the Pfizer BioNTech vaccine (Italian cohort 90.5%, Lebanese cohort 96.7%). Few subjects received AstraZeneca/Va (Italian cohort 7.8%, Lebanese cohort 1.4%) or other vaccines (Italian cohort 1.7%, Lebanese cohort 2.1%). A similar trend was recorded for the 2nd dose, with most subjects receiving Pfizer BioNTech vaccine (Italian cohort 96.7%, Lebanese cohort 98.3%), and a minority of subjects receiving AstraZeneca/Va (Italian cohort 1.3%, Lebanese cohort 0.3%) or other vaccines (Italian cohort 1.3%, Lebanese cohort 1.7%). In the Italian cohort, the rate of subjects reporting symptoms following the 1^st^ dose was higher for AstraZeneca, as compared to Pfizer BioNTech vaccine or others. The rate of post-vaccinal symptoms was comparable among different vaccines following the administration of the 2nd dose. In the Lebanese cohort, comparable rates of symptoms were recorded following both the 1st and the 2nd dose when subjects were divided according to type of vaccine.

### Italian cohort

In total, 1975 Italian individuals aged 42.9 ± 16.8 years received the 1st dose, with 1483 (75.1%) symptomatic subjects with a mean of 1.9 ± 1.7 symptoms. Following the administration of the 2nd dose, the prevalence of symptomatic subjects and the number of reported symptoms increased significantly to 79.8% and 2.9 ± 2.0 respectively. With respect to sex, females were significantly (p < 0.0001) more represented (64.5%), younger, with higher symptom prevalence and number of symptoms. The sex difference persisted after the 2^nd^ dose.

### Lebanese cohort

In total, 822 Lebanese subjects aged 32.5 ± 15.9 yrs. received the 1st dose. This group was significantly younger than the Italian cohort. Among this subgroup, 37.3% were symptomatic and reported a mean of 5.1 ± 3.8 symptoms. After the 2nd dose, the rate of symptomatic subjects increased to 59.7% but the number of reported symptoms remained comparable. With respect to sex, females were significantly (p < 0.0001) younger, with higher symptom prevalence. This sex difference persisted after the 2nd dose, with invariably and significantly higher prevalence of reported symptoms in females than males (p < 0.0001).

### Comparison between Italian and Lebanese cohorts

**Fig. 1:**
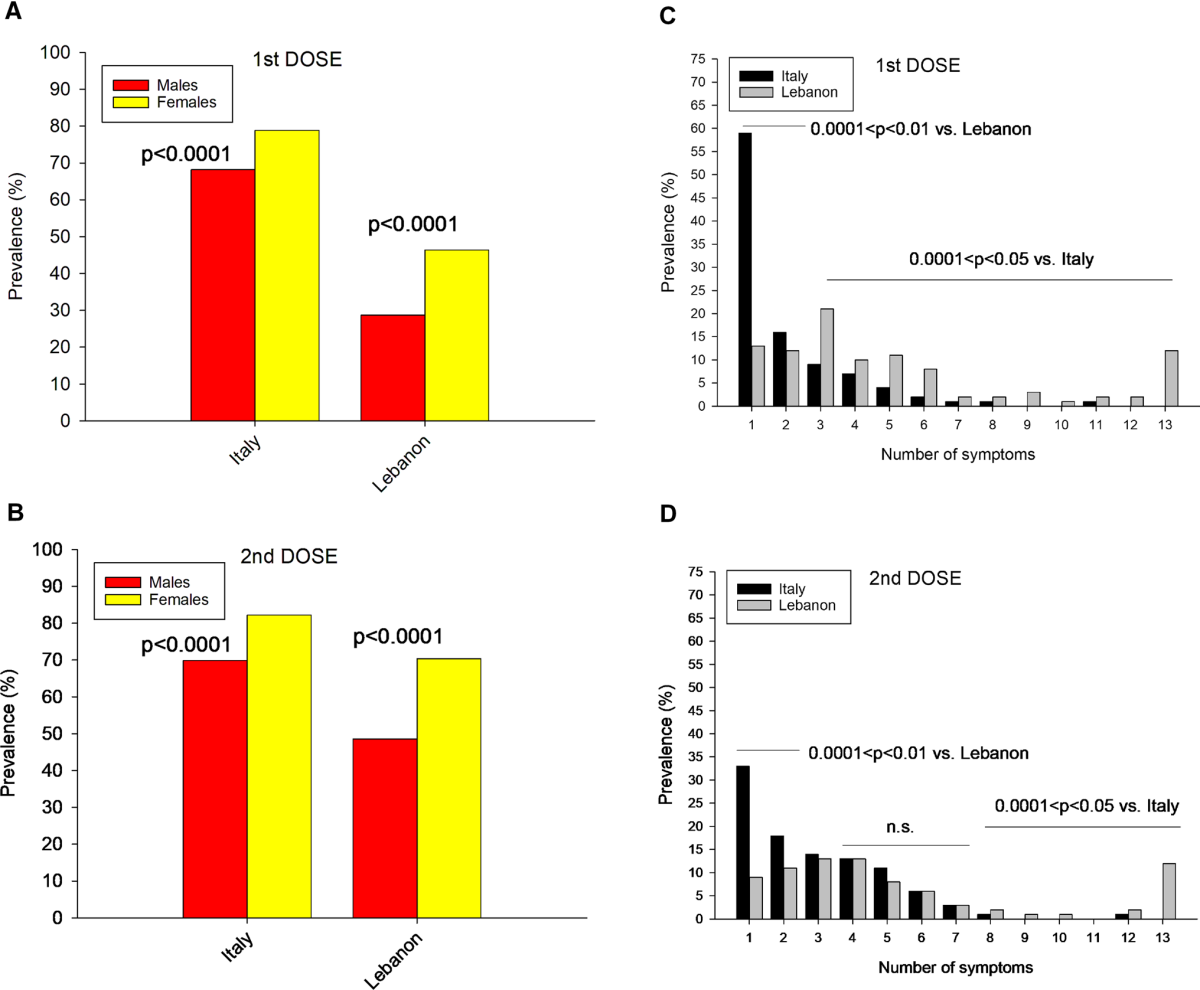
**A** Prevalence of symptomatic subjects according to living area and sex distribution after 1st dose of vaccine. **B** Prevalence of symptomatic subjects according to living area and sex after 2nd dose of vaccine. **C** Prevalence of symptomatic subjects according to living area and number of symptoms after 1st dose of vaccine **D** Prevalence of subjects according to living area and number of symptoms after 2nd dose of vaccine; *ns*: not significant (Chi-square test)

As shown in Table 1, Italian subjects were significantly (0.0001 < p < 0.01) older, had higher symptom prevalence but lower number of reported symptoms than Lebanese subjects. When subjects were grouped according to sex, more females than males received both vaccine doses in the Italian but not in the Lebanese cohort. In both cohorts, females were significantly younger than males (0.0001 < p < 0.03). As shown in Fig. 1A, B, the prevalence of symptomatic subjects was invariably higher in women after both doses and in both cohorts. The prevalence of subjects reporting one up to 13 symptoms after each vaccine dose is shown in Fig. 1C, D. Whereas most Italian subjects reported only one symptom (60%), many Lebanese subjects reported more than one symptom after each dose.

Characteristics of participants according to cohort, vaccine doses, sex, and geriatric age (i.e., < and ≥ 65 yrs.) are depicted in Table 2.

**Table 2:** Characteristics of the 2797 participants according to living area, vaccine doses, sex distribution, and geriatric age

	Males (N = 702)		Females (N = 1273)	
	< 65 yrs	≥ 65 yrs	< 65 yrs	≥ 65 yrs
Italian Cohort				
1st dose N (%)	601	101	1170	103
Age (yrs)	40.5 ± 13.3	72.8 ± 8.0	38.2 ± 11.9	80.8 ± 9.7
Symptomatic N (%)	433 (72.0%)*^,^,a^	46 (45.5%)*	965 (82.5%)^#,^,a^	39 (37.9%)^#^
N. of symptoms	1.7 ± 1.3	1.9 ± 1.7	2.2 ± 1.8^a^	1.5 ± 0.8
2nd dose	538 (89.5%)	93 (92.1%)	1026 (87.7%)	101 (98.1%)
Age (yrs)	41.0 ± 13.5	72.6 ± 7.8	38.0 ± 12.0	80.8 ± 9.7
Symptomatic N (%)	398 (74.0%)*	43 (46.2%)*	885 (86.3%)^#^	41 (40.6%)^#^
N. of symptoms	2.5 ± 1.9^^,a^	2.3 ± 2.4	3.3 ± 2.1^#,^^^^^,a^	1.8 ± 1.1^#^

Lebanese cohort	Males (N = 421)		Females (N = 401)	
	< 65 yrs	≥ 65 yrs	< 65 yrs	≥ 65 yrs
1st dose	378	43	362	39
Age (yrs)	29.5 ± 10.4	72.4 ± 3.9	26.5 ± 6.6	72.6 ± 6.8
Symptomatic N (%)	112 (29.6%)^a,$^	9 (20.9%)	165 (45.6%)^a,$^	21 (53.8%)
N. of symptoms	4.9 ± 3.9	1.7 ± 2.5	5.4 ± 4.0^e^	3.3 ± 2.3
2nd dose	321 (84.9%)	33 (76.6%)	331 (91.4%)	37 (94.9%)
Age (yrs)	28.9 ± 10.0	72.6 ± 3.4	26.4 ± 6.5	71.8 ± 6.6
Symptomatic N (%)	161 (50.1%)^$^	11 (33.3%)	238 (71.9%)^$^	21 (56.7%)
N. of symptoms	4.7 ± 3.9^a^	1.9 ± 2.5	5.5 ± 3.8^a^	3.6 ± 2.2

Data are expressed as number (N), mean ± SD, percentages (%). Difference between means were tested by Mann–Whitney test. Different percentages were tested by chi‐square test

*p < 0.01: differences between age groups for males, Italy;

^#^: p < 0.01: differences between age groups for females, Italy;

^: p < 0.01: differences between sexes in the Italian cohort;

^$^p < 0.01: differences between 1st vs 2nd dose in the Lebanese cohort;

^a^p < 0.01: differences between Italian and Lebanese cohorts;

In the Italian cohort, the prevalence of symptomatic subjects decreased at older age in both sexes (from 72.0 to 45.5% in males and from 82.5 to 37.9% in females, p < 0.0001 for both) after the 1st dose. In addition, the prevalence of symptomatic subjects was higher (p < 0.0001) in adult females than in adult males (< 65 years). Such differences persisted after the administration of the 2nd dose. Adult females had significantly more symptoms than older females and adult males (p < 0.0001).

In the Lebanese cohort, the number of older subjects was small (after 1^st^ dose 82/822 = 10% aged ≥ 65). The prevalence of symptomatic subjects in adults in both sexes was lower than that observed in the Italian group (Lebanese males 29.6% vs Italian males 72.0%, p < 0.0001; Lebanese females 45.6% vs Italian females 82.5%, p < 0.0001). At older ages, the prevalence of symptomatic subjects remained comparable in both sexes.

Notably, after the 2nd dose, the prevalence of symptomatic subjects increased in adults (from 29.6 to 50.1% in males, and from 45.6 to 71.9% in females, p < 0.0001) and tended to increase at older age only in males. The number of reported symptoms was in general higher than that found in the Italian cohort at any age and in both sexes (p < 0.0001).

**Fig. 2:**
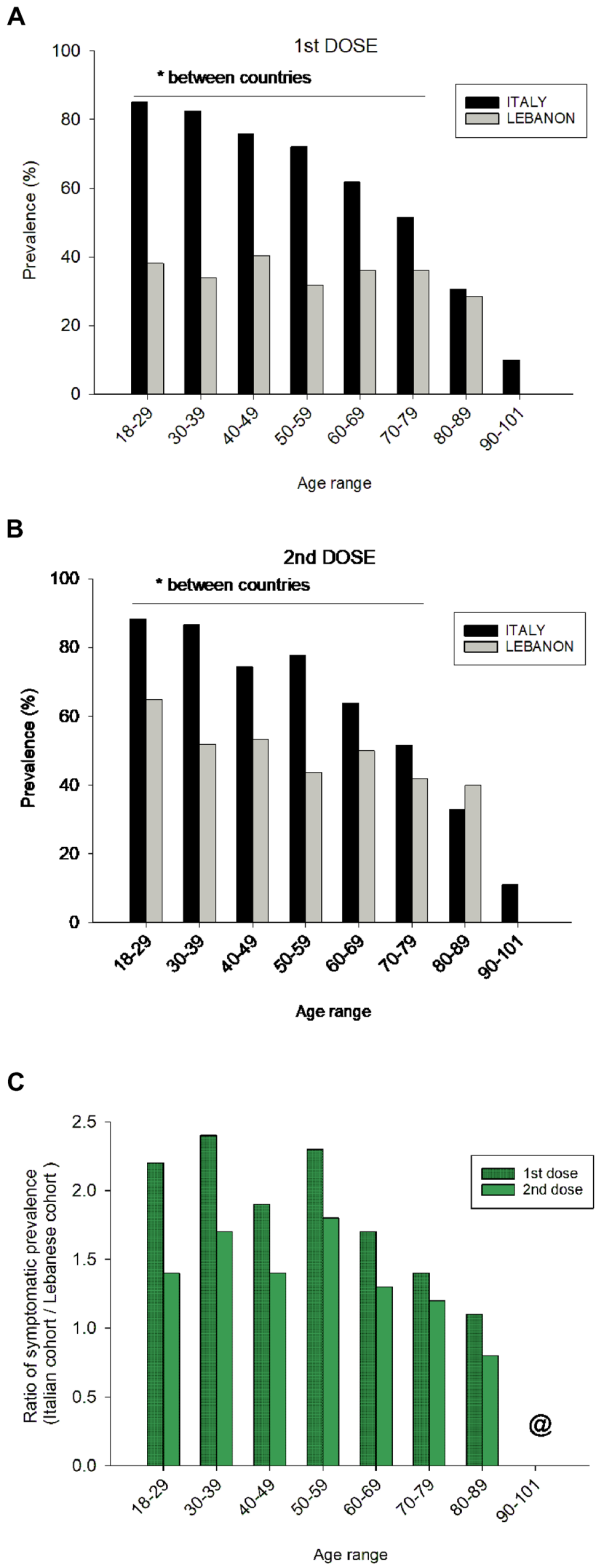
**A** Prevalence of symptomatic subjects according to living area and age class after 1^st^ dose of vaccine. **B** Prevalence of symptomatic subjects according to living area and age class after 2^nd^ dose of vaccine. **C** Ratio of symptomatic prevalence (Italian cohort / Lebanese cohort) according to age class after 1st and 2nd dose of vaccine. ^@^In the age class 90–101 symptomatic individuals were absent in the Lebanese cohort

Since the Lebanese cohort was younger than the Italian cohort, we further explored the prevalence of subjects reporting post-vaccinal symptoms in both cohorts divided according to eight age classes, i.e., 18–29, 30–39, 40–49, 50–59, 60–69, 70–79, 80–89, and 90–101 years. Data stratified by cohort, vaccine dose, and age groups are provided in Fig. 2A–C. The prevalence of symptoms decreased progressively with the age class in the Italian cohort, but not in the Lebanese cohort. The prevalence of subjects reporting post-vaccinal symptoms was higher in the Italian than in the Lebanese cohort at all age classes up to 70–79 years, and after both doses (Fig. 2A, B). In addition, the prevalence of symptomatic subjects following the administration of the 1^st^ dose was about twice in the Italian, as compared with the Lebanese cohort, from the age class 18–29 to 60–69 years. This difference decreased progressively in subjects older than 70 years. The ratios between the symptomatic rates recorded in the two cohorts (Fig. 2C) were lower following the 2nd dose. However, the rate of post-vaccinal symptoms still remained about 1.5-folds higher in the Italian, than in the Lebanese cohort up to the age class 50–59 years, progressively decreasing in older ages.

**Fig. 3:**
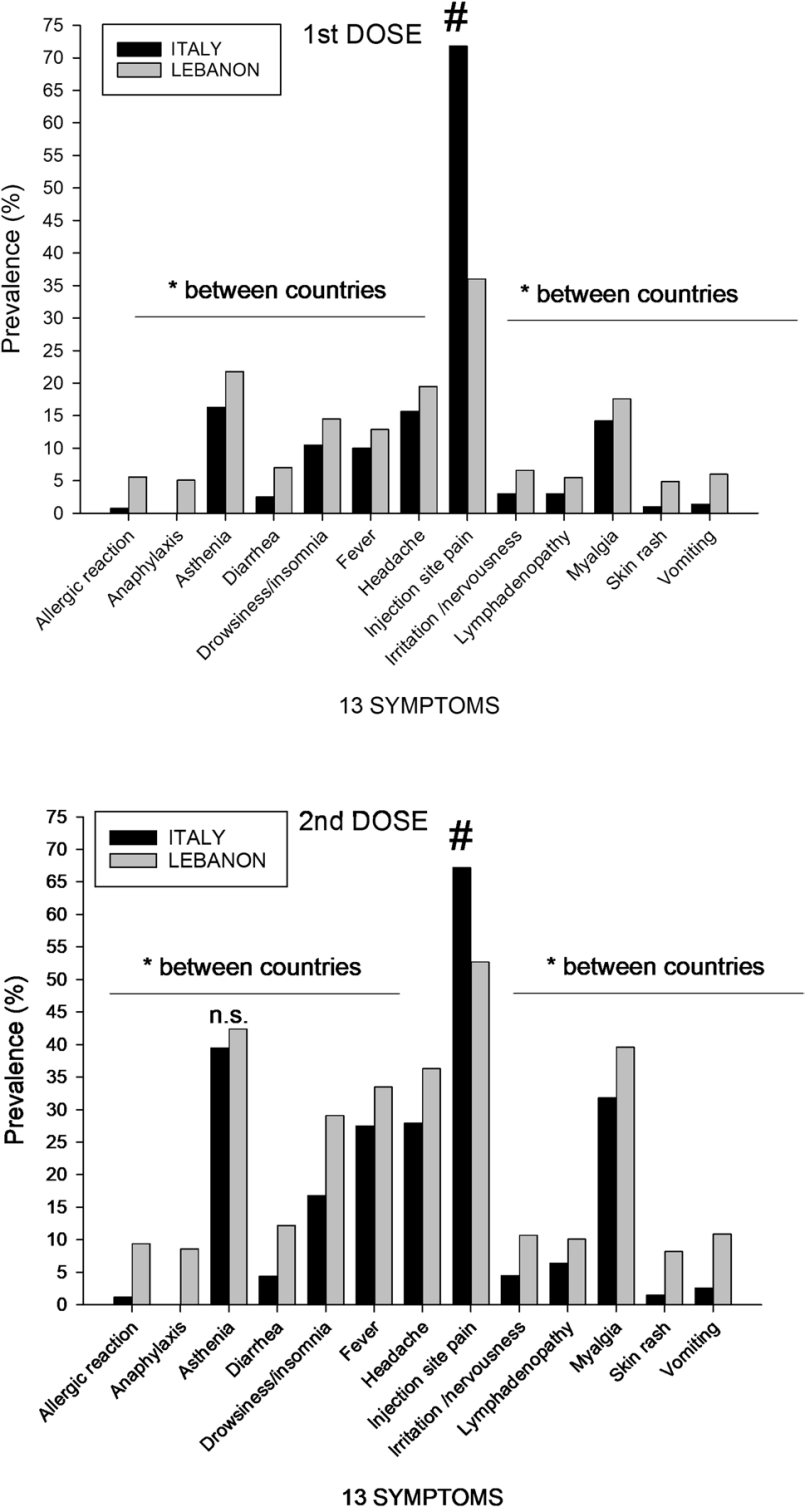
Prevalence of 13 symptoms in Italian and Lebanese subjects in response to 1st and 2nd dose of vaccine. Differences tested by Chi-square test. Asterisk indicates (*) significantly higher than in Italian subjects (0.0002 < p < 0.05); (#) significantly higher than in Lebanese subjects p < 0.0001; *ns*, not significant

**Fig. 4:**
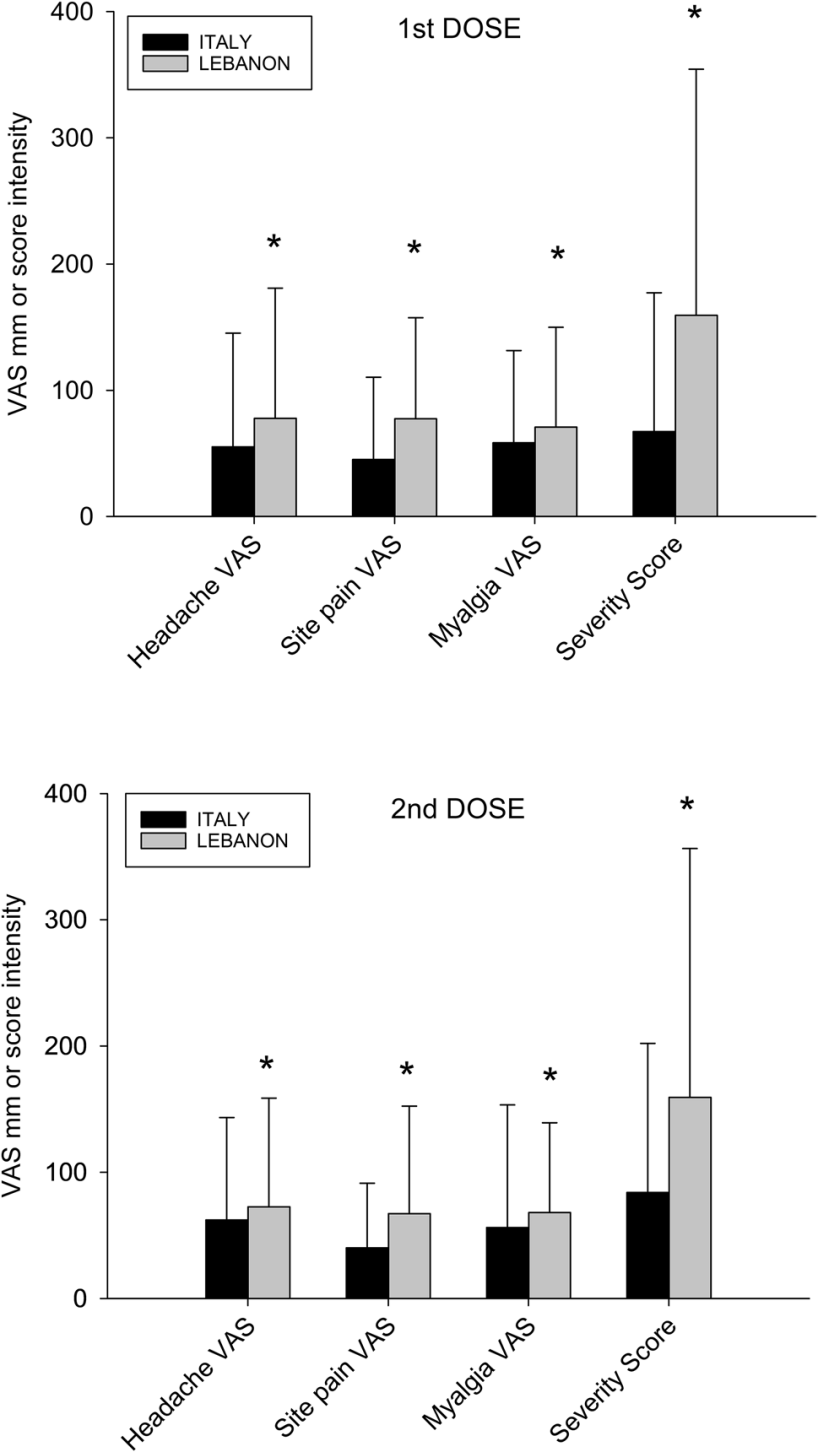
Severity of selected symptoms (VAS) and severity score (intensity) in Italian and Lebanese individuals after 1st and 2nd dose of vaccine. Asterisk indicates (*) significantly higher values than in Italian subjects (0.0002 < p < 0.05). Differences tested by Mann–Whitney

We further explored the effect of each dose of vaccine on the prevalence of 13 symptoms as well as the intensity (VAS) of selected symptoms according to living area and sex (Table 3, Fig. 3 and 4).

**Table 3:** Type of symptoms reported after 1^st^ and 2^nd^ doses of vaccine according to living area and sex distribution in 2797 subjects

1st dose	Italian cohort (N = 1975)				Lebanese cohort (N = 822)			
	All, N (%)	Males	Females	*p*	All, N (%)	Males	Females	*p*
		N = 702	N = 1273			N = 421	N = 401	
Symptoms								
Allergic reaction	16 (0.8%)	2 (0.3%)	14 (1.1%)	0.053	46 (5.6%)	17 (4.0%)	29 (7.2%)	0.046
Anaphylaxis	0 (0%)	0 (0%)	0 (0%)	NS	42 (5.1%)	16 (3.8%)	26 (6.5%)	0.080
Diarrhea	49 (2.5%)	9 (1.3%)	40 (3.1%)	0.01	58 (7.1%)	35 (8.3%)	23 (5.7%)	0.15
Fever	197 (10.0%)	51 (7.3%)	146 (11.5%)	0.003	106 (12.9%)	41 (9.7%)	65 (16.2%)	0.006
Headache	311 (15.7%)	69 (9.8%)	242 (19.8%)	< 0.0001	152 (19.5%)	59 (14.0%)	93 (23.2%)	0.0007
Injection site pain	1418 (71.8%)*	460 (65.5%)	958 (75.3%)	< 0.0001	296 (36.0%)*	116 (27.6%)	180 (44.9%)	< 0.0001
Insomnia	207 (10.5%)	40 (5.7%)	167 (13.1%)	< 0.0001	119 (14.5%)	45 (10.7%)	74 (18.5%)	0.002
Irritability/nervousness	59 (3.0%)	12 (1.7%)	47 (3.7%)	0.01	54 (6.6%)	20 (4.8%)	34 (8.5%)	0.03
Lymphadenopathy	60 (3.0%)	11 (1.4%)	49 (3.8%)	0.001	45 (5.5%)	17 (4.0%)	28 (7.0%)	0.06
Myalgia	281 (14.2%)	71 (10.1%)	210 (16.5%)	0.001	145 (17.6%)	58 (13.8%)	87 (21.7%)	0.003
Skin rash	20 (1%)	4 (0.6%)	16 (1.3%)	0.14	40 (4.9%)	17 (4.0%)	23 (5.7%)	0.26
Vomiting	28 (1.4%)	6 (0.9%)	22 (1.7%)	0.11	49 (6.0%)	18 (4.3%)	31 (7.7%)	0.04 0.005
Weakness	323 (16.3%)	83 (11.8%)	240 (18.9%)	0.0001	179 (21.8%)	75 (17.8%)	104 (25.9%)	
Headache intensity (VAS)	55.3 ± 88.2	36.5 ± 38.2	60.6 ± 98.0	0.02	77.9 ± 106.0	62.7 ± 95.2	88.9 ± 112.8	0.04
Site pain Intensity (VAS)	45.3 ± 64.0	35.6 ± 51.5	49.9 ± 68.1	< 0.0001	77.5 ± 80.9	79.7 ± 82.9	77.9 ± 79.2	0.7
Myalgia Intensity (VAS)	58.5 ± 72.1	38.1 ± 37.9	65.2 ± 79.7	0.02	70.9 ± 79.5	68.1 ± 89.9	72.9 ± 71.8	0.1
Severity Score (I)	67.3 ± 111.7	46.1 ± 65.7	77.3 ± 126.7	< 0.0001	159.4 ± 201.5	153.6 ± 207.9	163.3 ± 197.8	0.45

2nd dose	N = 1758	N = 631	N = 1127		N = 722	N = 354	N = 368	
Allergic reaction	22 (1.2%)		16 (1.4%)	0.4	68 (9.4%)	24 (6.8%)	44 (12.0%)	0.02
Anaphylaxis	0	0	0	NS	62 (8.6%)	21 (5.9%)	41 (11.1%)	0.01
Diarrhea	78 (4.4%)	20 (3.2%)	58 (5.1%)	0.053	88 (12.2%)	32 (9.0%)	56 (15.2%)	0.01
Fever	485 (27.5%)	123 (19.5%)	362 (32.1%)	< 0.0001	242 (33.5%)	83 (23.4%)	159 (43.2%)	< 0.0001
Headache	490 (27.9%)	112 (17.7%)	378 (33.5%)	< 0.0001	262 (36.3%)	106 (29.9%)	156 (42.4%)	0.0005
Injection site pain	1181 (67.2%)	377 (59.7%)	804 (71.3%)	< 0.0001	381 (52.7%)	147 (41.5%)	234 (63.6%)	< 0.0001
Insomnia	296 (16.8%)	59 (9.4%)	237 (22.7%)	< 0.0001	210 (29.1%)	78 (22.0%)	132 (35.9%)	< 0.0001
Irritability/Nervousness	80 (4.5%)	25 (4.0%)	55 (4.9%)	0.37	77 (10.7%)	31 (8.8%)	46 (12.5%)	0.1
Lymphadenopathy	113 (6.4%)	28 (4.4%)	85 (7.5%)	0.01	73 (10.1%)	23 (6.5%)	50 (13.6%)	0.002
Myalgia	559 (31.8%)	139 (22.0%)	420 (37.3%)	< 0.0001	286 (39.6%)	107 (30.2%)	179 (48.6%)	< 0.0001
Skin rash	26 (1.5%)	6 (1.0%)	20 (1.8%)	0.17	59 (8.2%)	22 (6.2%)	37 (10.1%)	0.06
Vomiting	46 (2.6%)	7(1.1%)	39 (3.5%)	0.003	79 (10.9%)	24 (6.8%)	55 (14.9%)	0.0004
Weakness	695 (39.5%)	189 (30.0%)	506 (44.9%)	< 0.0001	306 (42.4%)	110 (31.1%)	196 (53.3%)	< 0.0001
Headache intensity (VAS)	62.3 ± 81.9	49.0 ± 46.6	66.2 ± 87.5	0.09	72.7 ± 87.4	72.0 ± 110.2	73.2 ± 83.7	0.2
Site pain Intensity (VAS)	40.3 ± 51.5	32.4 ± 40.8	43.9 ± 56.7	< 0.0001	67.4 ± 87.8	61.9 ± 78.8	69.0 ± 93.3	0.04
Myalgia Intensity (VAS)	56.4 ± 99.3	43.9 ± 47.2	60.5 ± 71.7	0.001	68.2 ± 71.0	58.2 ± 66.2	74.2 ± 72.2	0.002
Severity Score (II)	84.1 ± 119.9	56.8 ± 79.8	97.0 ± 130.9	< 0.0001	159.4 ± 195.1	145.9 ± 186.2	168.3 ± 197.9	0.01

Data are expressed as number (N), percentages (%), mean ± SD. N: Number of subjects, VAS: Visual analogue scale. Differences of percentages between sexes: Chi-square test. Comparison of symptoms intensity and score between sexes: Mann–Whitney test

The detailed analysis is reported in Table 3, and shows that after the administration of the 1st dose, and overall, the prevalence of reported symptoms ranged from 0 to 71.8% in the Italian cohort and from 4.9 to 36.0% in the Lebanese cohort. Most subjects reported pain at the injection site, more evident in the Italian cohort (71.8% vs. Lebanese cohort 36.0%, p < 0.0001). All other symptoms had greater prevalence in the Lebanese- than the Italian cohort (Fig. 3).

Since the prevalence of injection site pain was the most relevant difference between the Lebanese and Italian cohorts, we also calculated the rate of symptomatic subjects in the two cohorts excluding subjects who reported injection site pain as the unique symptom (Supplementary Table S3). In this case, the prevalence of symptomatic subjects was comparable between the two cohorts.

In both cohorts we observed a sex difference with invariably higher prevalence of symptomatic females than males for all symptoms but allergic reaction, skin rash, and vomiting in the Italian cohort (0.0001 < p < 0.01 between sexes) and anaphylaxis, diarrhea, lymphadenopathy, and skin rash in the Lebanese cohort (0.0001 < p < 0.046 between sexes) (Table 3). Likely, the sex difference was less evident among Lebanese due to the younger age of subjects.

The analysis of the severity of selected symptoms yielded higher values in females than in males for headache, site pain, and myalgia in Italian subjects, and for headache in Lebanese subjects. A further analysis of each symptom according to living area and sex is provided in Table 3 and Fig. 4.

After the administration of the 2nd dose, and overall, the prevalence of reported symptoms was 0–67.2% in the Italian cohort and 8.2–52.7% in Lebanese cohort. Similarly to the 1st dose, most subjects reported pain at the injection site (Italian cohort 67.2%, Lebanese cohort 52.7%, p < 0.05). The prevalence of all symptoms was higher after the 2nd than the 1st dose in both cohorts and in the female sex.

We observed a sex difference with invariably higher prevalence of symptomatic females than males for all symptoms with exception of allergic reaction, diarrhea, irritation/nervousness, and skin rash, in the Italian cohort (0.0001 < p < 0.016) and irritation/nervousness, and skin rash in the Lebanese cohort (0.0001 < p < 0.04). With respect to the 1^st^ dose, the sex difference was comparable between the two cohorts.

**Fig. 5:**
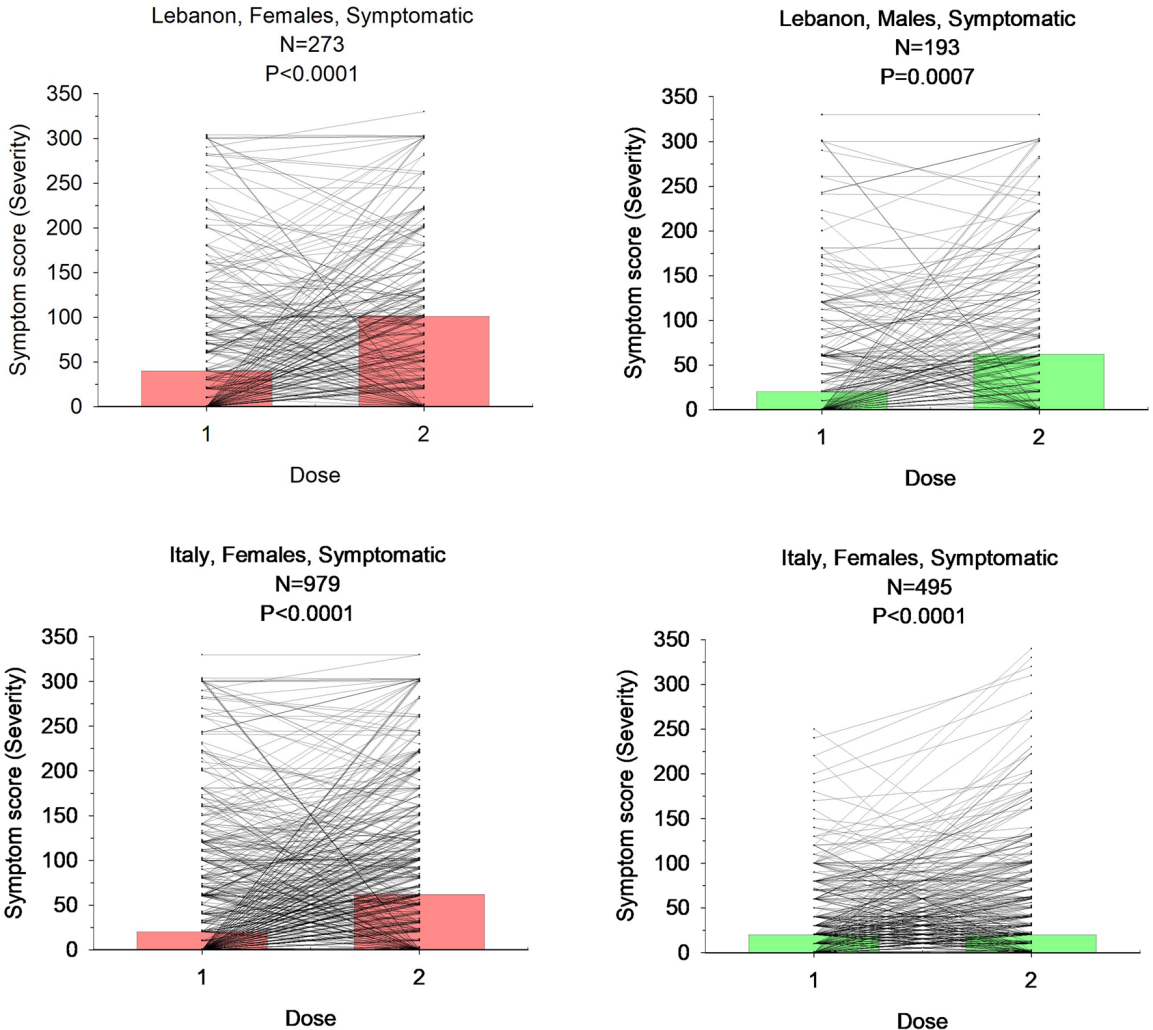
Individual changes of symptom severity score in Lebanese and Italian individuals after 1st and 2nd dose of vaccine, according to sex distribution. Coloured bars indicate medians. Differences tested by Mann–Whitney

The analysis of the severity of symptoms yielded higher values in females than in males for headache, injection site pain, and myalgia in the Italian cohort, and for injection site pain and myalgia in the Lebanese cohort (Table 3 and Fig. 4). The analysis of individual changes of symptom score intensity is depicted in Fig. 5. In both living areas the overall score increased after the 2nd dose and became more evident in Lebanese than in Italians.

Concerning the final judgement about vaccination, 98.2% and 99.1% of Italian and Lebanese subjects would have repeated the procedure, irrespective of the appearance of symptoms after vaccine administration.

## Discussion

We had the chance to compare two free-living Italian and Lebanese cohorts who received the 1st dose of anti-COVID-19 vaccine and in most cases, a 2nd dose, i.e., mainly Pfizer vaccine. By using a tailored questionnaire specifically adapted to the target populations by questions and languages, we detected several distinct differences in self-reported symptoms according to countries, vaccine dose, sex, and age classes.

According to the World Health Organisation (WHO), COVID-19 vaccines have played a pivotal role to slow down infection rates, to prevent severe symptoms and to decrease the risk of hospitalization and mortality. However, anti-COVID-19 vaccines can induce symptoms which, in some cases, resemble those of a mild COVID-19 infection [16, 17]. We speculated that qualitative and quantitative differences might exist between two population living in the Mediterranean basin, based on ethnic and sex differences.

We found that about two-thirds of Italian participants described symptoms after the 1^st^ dose. In contrast, one third of the Lebanese individuals were symptomatic after vaccination, despite the absolute number of symptoms was higher in Lebanese cohort. In addition, the prevalence of symptoms after vaccination was higher in females from both cohorts.

As compared with the Italian cohort, Lebanese subjects reported a significantly lower prevalence of injection site pain following both doses of vaccine. The prevalence of this symptom in the Lebanese cohort was also markedly lower than that recorded in other European cohorts, which ranges from 70 to 80% of subjects [18]. Of note, however, findings from the present study (i.e., injection site pain in 36% and 41.5% of Lebanese subjects following the 1st and the 2nd dose of Pfizer vaccine, respectively) are comparable to those from a previous large retrospective survey in Lebanese subjects, reporting injection site pain in 33.1% of subjects after the 1st dose, and in 46.6% after the 2nd dose [19]. The different prevalence of the injection site pain in European and Lebanese subjects might be explained, at least in part, by a different pain perception threshold in the two ethnic groups, which could be the real difference among the two cohorts. In fact, we also calculated the prevalence of symptomatic subjects in the two cohorts excluding subjects who reported injection site pain as the unique symptom. In this case, the prevalence of symptomatic subjects was comparable between the Italian and Lebanese cohort, although maintaining the sex-dependent effect. A further possible explanation for the different prevalence of injection site pain could be, in the present study, the different mean age of subjects enrolled in the two cohorts. In fact, in our survey Lebanese were younger than Italians and this dissimilarity might affect the different pain perception threshold observed in the two cohorts. Moreover, the site injection pain might depend on several anatomical and physiological patterns (i.e., lesion in the deltoid muscle, muscle edema, increased membrane permeability of small blood vessels), which can vary with age, sex, ethnicity [20].

When all subjects were stratified according to age classes, the rate of symptomatic subjects was systematically higher in the Italian than in the Lebanese cohort up to the age class 70–79 years, and became comparable only in subjects aged 80 or more years, with obvious limitations within this age range. Our study is in line with previous reports showing that the frequency and the severity of post-vaccine reactions decreases with age [21]. However, in our study this difference occurred only in the Italian but not in the Lebanese cohort, pointing to a potential role of ethnic or genetic factors.

The observed higher symptom prevalence in Italian subjects compared to Lebanese subjects might be also attributed to the higher number of female participants in the Italian cohort (64.5%). This group reported a significant and remarkable increase of symptoms compared to males.

Regarding symptoms, the most frequent in response to both doses were pain at the injection site, weakness, and headache in both sexes and cohorts. These results are in line with data [16, 18, 22, 23] from different geographical area i.e., USA, UK, and Saudi Arabia, reporting that pain at the injection site was the most abundant “local” side effects and headache, weakness/fatigue, and myalgia were the most prevalent “systemic” side effects. The findings are not closely related to age per se and living area. The centre for diseases control and prevention (CDC) reported that the side effects in response to Pfizer vaccine were higher after the 2nd dose [22]. Accordingly, in both countries, the prevalence and intensity of each symptom as well as the overall severity score increased after the 2nd dose, particularly in the Lebanese cohort. Mohammed et al. [22] in Saudi Arabia showed that Pfizer/BioNTech vaccination is safe, has no reported anaphylaxis or serious events, and that local pain and fatigue are the most common reported side effects which are mild to moderate in nature with a regressive course. More side effects were experienced after the 2nd dose, as compared with the 1st. The significant predictors of side effects were the female sex and a history of allergies. In Poland, Andrzejczak-Grzadko et al. [24] compared Astra Zeneca with Pfizer vaccine and reported that among those vaccinated with the first Pfizer dose, vaccine reactions were described in 93.9% of respondents. The 2nd dose of the Pfizer vaccine caused post-vaccinal reactions in 54.8% of respondents (more adverse reactions) plus 15.8% (fewer adverse reactions). Same side effects were experienced in 29.4% of subjects after the 1st and 2nd doses of the Pfizer vaccine. Thus, also in the present study, we conclude that side effects after Pfizer vaccination are not rare. A higher prevalence of symptoms after vaccines seems to occur in females than in males [16, 22]. Apparently, this was the case in both Italian and Lebanese subjects for both prevalence and intensity as well as the severity score in females.

The present study was not designed to assess the serological response of enrolled subjects. Only questionnaires were employed and the timeframe for self-reported symptoms was restricted to seven days after vaccination in subjects without previous COVID-19 infection. Nevertheless, in a large study, Cangemi et al. [25] reported that the anti-Spike IgG were inversely associated with age, and a reduction of more than 82% was directly associated with male sex.

The sex-dependent effect occurs in adult participants (< 65 years), while differences were not observed between two sexes in the geriatric group (≥ 65 years) in both cohorts. Likely, less geriatric subjects experienced symptoms in response to anti-COVID-19 vaccines. According to the food and drug administration (FDA), younger individuals experienced more pronounced side effects in response to anti-COVID-19 vaccine than older subjects [26]. This finding is likely due to the immunosenescence that causes a decline of the efficacy of the immune system, leading to increased vulnerability to COVID-19 and decreased responses to vaccination [27].

Sex differences can parallel variable endocrine [28] and/or immunological pathways [29] in both males and females. Estrogens play a protective role against the COVID-19 infection through promoting anti‐inflammatory Th2 responses and inhibiting the pro‐inflammatory innate immune response [30]. Conversely, an in vivo animal study shows that the low level of estrogens following ovariectomy or exposure to estrogen receptor antagonist increases mortality after SARS‐CoV infection [31]. These results were confirmed in a study which found a higher severity of COVID-19 symptoms in postmenopausal females (a group with low level of estrogens) and in young females that had not taken any combined oral contraceptive pill, as compared to females taking pills containing estrogens [32]. This prevalence may be due to the high innate immune system activity in females, mediated by Toll-like receptors, retinoic acid-inducible gene I-like receptors, and nucleotide oligomerization domain-like receptors, and leads to the increase of type 1 interferon (IFN) and inflammatory cytokines (IL-1, TNFs) [33].

In addition to immunological and hormonal factors, social and ethnic factors may play a critical role in explaining the differential outcomes in the two sexes.

The strength of the present study relies on the simultaneous comparison of two different cohorts with profound cultural and health service differences living in the Mediterranean basin. Despite the questionnaires were anonymous, we did show distinct outcomes in Italian and Lebanese subjects, also evident at different age classes and with several sex-specific profiles.

Nevertheless, our study had some limitations. In Italy, the sex and age proportions were not perfectly distributed. This is because most healthcare workers including doctors and nurses, are women (~ 70%) aged 35–64 years as reported by the Italian ministry of health in 2019 [34]. Similarly, in Lebanon, the mean age was lower and most healthcare workers were adult women [35]. Ongoing research is trying to match the populations more closely to confirm the results of the present study.

We found a significantly higher rate of “anaphylaxis” in the Lebanese- than in the Italian cohort. This finding, however, should be interpreted with caution due to a possible misunderstanding in the Arabic translation of the questionnaire. Since there is not a specific term for “anaphylaxis” in Arabic, in almost all translations websites (including google translator) anaphylaxis is translated as "حساسية مفرطة, which literally means “excessive allergy”. The questionnaire was designed to ensure that all medical terms defining symptoms should be clear and simple for the majority of people, and we also offered the questionnaire in English language (see supplementary material Table S1) to limit possible terminological misunderstandings. However, a terminological misinterpretation cannot be excluded and this can be considered a limitation of the present study. Further confirmation of the exact prevalence of post-vaccinal anaphylaxis in the Lebanese cohort is therefore needed.

In conclusion, the present study brings novel insights into the impact of COVID-19 vaccines as post-vaccinal symptoms in free-living populations. Results underscore the importance of considering not only age but also geographical areas and sex-related effects. To our knowledge, this is a first study exploring self-reported symptoms after COVID-19 vaccination from two different cohorts in the Mediterranean area at the same time. Further investigations need to address this topic with respect to clinical and social aspects.

## Supplementary Information

Below is the link to the electronic supplementary material.

Supplementary file1 (DOCX 82 KB)

## Data Availability

The datasets generated during and/or analysed during the current study are available from the corresponding author on reasonable request.
