# Supplementary material for: Self-reported symptoms after COVID-19 vaccination. Distinct sex, age, and geographical outcomes in Lebanese and Italian cohorts
Source: Intern Emerg Med. 2023 Jun 15;18(5):1463–75. doi: 10.1007/s11739-023-03321-9 (PMC10412474; doi:10.1007/s11739-023-03321-9)
Supplement: Supplementary file 1 — Supplementary file1 (DOCX 82 KB) [file 11739_2023_3321_MOESM1_ESM.docx]

**TABLE S1**

**ITALIAN SURVEY**

Clinica Medica "Augusto Murri"

Informazioni su vaccinazione anti-Covid ed eventuali effetti collaterali.

Vi chiedo qualche minuto del vostro prezioso tempo per la compilazione del questionario per valutare differenze relative al sesso. La compilazione è anonima ed i dati saranno trattati con la più assoluta confidenzialità. Si ringrazia per la collaborazione. Prof. Piero Portincasa

Email*

Il tuo indirizzo email

Sesso*

Uomo

Donna

Età*

La tua risposta

Professione / Mansione*

medico

infermiere

oss / pulitore

tecnico di laboratorio / biologo

dottorando

studente

amministrativo

Forze Armate

Personale scolastico

Pensionato/a

Altro:

Prima dell'esecuzione del vaccino anti-covid, ha mai presentato una reazione avversa a vaccini?*

SI

NO

Se SI, ricorda a quale vaccino?

La tua risposta

Se SI, quale tipo di reazione avversa?

dolore nel sito di iniezione

reazione allergica-orticarioide

anafilassi

febbre (> 37°C)

diarrea

vomito

astenia / malessere

mialgie

eruzione cutanea

sonnolenza/insonnia

irritabilità/nervosismo

cefalea

linfoadenopatia

altro

Quale vaccino anti-Covid Le hanno somministrato?*

Pfizer-BioNTech (Comirnaty)

Moderna (mRNA 1273)

AstraZeneca

Altro:

Ha eseguito la somministrazione della prima dose di vaccino anti-covid?*

SI

NO

Ha avuto qualche tipo di reazione?*

SI

NO

Se SI, quale?

durata (0-12h)

durata (12-24h)

Giorno 1

Giorno 2

Giorno 3

Giorno 4

Giorno 5

Giorno 6

Giorno 7

dolore nel sito di iniezione

rossore nel sito di iniezione

gonfiore nel sito di iniezione

indurimento nel sito di iniezione

reazione allergica-orticarioide

anafilassi

febbre (>37°C)

diarrea

vomito

astenia / malessere

mialgia

eruzione cutanea

sonnolenza / insonnia

irritabilità / nervosismo

cefalea

Linfoadenopatia

dolore nel sito di iniezione

rossore nel sito di iniezione

gonfiore nel sito di iniezione

indurimento nel sito di iniezione

reazione allergica-orticarioide

anafilassi

febbre (>37°C)

diarrea

vomito

astenia / malessere

mialgia

eruzione cutanea

sonnolenza / insonnia

irritabilità / nervosismo

cefalea

Linfoadenopatia

In particolare, come descriverebbe l'intensità del sintomo dolore?

nessun dolore (VAS 0)

dolore molto lieve (VAS 1 - 2)

dolore lieve (VAS 3 - 4)

dolore moderato (VAS 5 - 6)

dolore forte (VAS 7 - 8)

dolore molto forte (VAS 9 - 10)

dolore nel sito di iniezione

mialgia

cefalea

dolore nel sito di iniezione

mialgia

cefalea

In caso di temperatura > 37 °C ...

SI

NO

Ha presentato una sola puntata febbrile?

la temperatura massima registrata è stata > 38 °C

Ha fatto ricorso ad antipiretici?

Ha presentato una sola puntata febbrile?

la temperatura massima registrata è stata > 38 °C

Ha fatto ricorso ad antipiretici?

Per il tipo di reazione avversa presentato...

SI

NO

Ha assunto farmaci ?

Ha fatto ricorso al MMG ?

Ha effettuato accesso al Pronto Soccorso?

Si è resa necessaria l'ospedalizzazione?

Ha assunto farmaci ?

Ha fatto ricorso al MMG ?

Ha effettuato accesso al Pronto Soccorso?

Si è resa necessaria l'ospedalizzazione?

Ha eseguito la somministrazione della seconda dose di vaccino anti-covid?*

SI

NO

In quale data le hanno somministrato la seconda dose di vaccino?

Data

Ha avuto qualche tipo di reazione?*

SI

NO

Se SI, quale?

durata (0-12 h)

durata (12-24 h)

Giorno 1

Giorno 2

Giorno 3

Giorno 4

Giorno 5

Giorno 6

Giorno 7

Dolore nel sito di iniezione

Rossore nel sito di iniezione

Gonfiore nel sito di iniezione

Indurimento nel sito di iniezione

Reazione allergica-orticarioide

Anafilassi

Febbre (>37°C)

Diarrea

Vomito

Astenia / Malessere

Mialgia

Eruzione cutanea

Sonnolenza / insonnia

Irritabilità / nervosismo

cefalea

Linfoadenopatia

Dolore nel sito di iniezione

Rossore nel sito di iniezione

Gonfiore nel sito di iniezione

Indurimento nel sito di iniezione

Reazione allergica-orticarioide

Anafilassi

Febbre (>37°C)

Diarrea

Vomito

Astenia / Malessere

Mialgia

Eruzione cutanea

Sonnolenza / insonnia

Irritabilità / nervosismo

cefalea

Linfoadenopatia

In particolare, come descriverebbe l'intensità del sintomo dolore?

nessun dolore (VAS 0)

dolore molto lieve (VAS 1 - 2)

dolore lieve (VAS 3 - 4)

dolore moderato (VAS 5- 6)

dolore forte (VAS 7 - 8)

dolore molto forte (VAS 9 - 10)

dolore nel sito di iniezione

mialgia

cefalea

dolore nel sito di iniezione

mialgia

cefalea

In caso di temperatura > 37 °C ...

SI

NO

Ha presentato una sola puntata febbrile?

La temperatura massima registrata è stata > 38 °C?

Ha fatto ricorso ad antipiretici?

Ha presentato una sola puntata febbrile?

La temperatura massima registrata è stata > 38 °C?

Ha fatto ricorso ad antipiretici?

Per il tipo di reazione avversa presentato...

SI

NO

ha assunto farmaci?

ha fatto ricorso al MMG?

ha effettuato accesso al PS?

si è resa necessaria l'ospedalizzazione?

ha assunto farmaci?

ha fatto ricorso al MMG?

ha effettuato accesso al PS?

si è resa necessaria l'ospedalizzazione?

Ha eseguito il dosaggio degli anticorpi anti SARS-COV2 SPIKE (IgG)?

SI

NO

Dopo quanti giorni dalla somministrazione della seconda dose?

La tua risposta

Inserisca il valore delle IgG anti SARS-COV-2 Spike

La tua risposta

Dopo aver ricevuto le dosi di vaccinazione, ripeterebbe la vaccinazione?

SI

NO

Commenti / suggerimenti

**ARABIC SURVEY**

Sezione 1 di 11

معلومات عن الآثار الجانبية المحتملة للقاح المضاد لفايروس كورونا

إذا كنت ممن أخذ اللقاح ضد كورونا ولم تصاب بمرض كورونا من قبل, هذه الإستمارة تتطلب بعض الدقائق من وقتك الثمين

لتجميع بعض المعلومات عن الآثار الجانبية المحتملة للقاح المضاض لفايروس كورونا للجنسين. هذه المعلومات سوف تبقى سرية, شكرا لتعاونكم...

Email address

Testo risposta breve

Gender/ الجنس

*

Age/ العمر

*

Testo risposta breve

Profession / Position/ المهنة / المنصب

*

Altro…

Before taking the covid vaccine, did you ever have an adverse reaction to any other vaccine?/هل سبق و تعرضت لمضاعفات جراء أخذ لقاح غير اللقاح المضاد  لفايروس كورونا؟

*

Dopo la sezione 1

Continua alla sezione successiva

Sezione 2 di 11

if yes...

Descrizione (facoltativa)

If YES, which vaccine?/إذا كان جوابك نعم,ما هو اللقاح؟

Testo risposta breve

If YES, what kind of adverse reaction?/إذا كان جوابك نعم,ما هي المضاعفات؟

Dopo la sezione 2

Continua alla sezione successiva

Sezione 3 di 11

Anti-COVID vaccine

Descrizione (facoltativa)

What anti-Covid vaccine did they give you?/ما هو اللقاح الذي أخذته

*

Altro…

Dopo la sezione 3

Continua alla sezione successiva

Sezione 4 di 11

Dose 1 /الجرعة الأولى

dose 1 of anti-COVID vaccine/الجرعة الأولى للقاح المضاد لفايروس كورونا

Did you have any kind of reaction after taking the first dose? هل حصلت مضاعفات بعد اخذ الجرعة الأولى؟

*

Dopo la sezione 4

Continua alla sezione successiva

Sezione 5 di 11

if yes...

Descrizione (facoltativa)

If so, which one?/إذا كان جوابك نعم, ما هي المضاعفات؟

(0-12ساعة)

(12-24ساعة)

Day 1/اليوم 1

Day 2/اليوم 2

Day 3/ اليوم 3

Day 4/ اليوم 4

Day 5/ اليوم 5

Day 6 االيوم 6

Day 7/ اليوم 7

pain at the injection site/ ألم مكان الحقنة

redness at the injection site/ إحمرار مكان الحقنة

swelling at the injection site/ ورم مكان الحقنة

induration at the injection site/ تصلب مكان الحقنة

allergic-urticarial reaction/ تحسس جلدي

anaphylaxis/ حساسية مفرطة

fever (> 37 ° C)/ارتفاع الحرارة أكثر من 37درجة مأوية

diarrhea/ إسهال

vomiting/ تقيئ

asthenia / fatigue/تعب و فقدانالقوة

muscle pain/ألم العضلات

rash/ طفح جلدي

sleepiness / insomnia/ النعاس او الأرق

irritability / nervousness/ التهيج او العصبية

headache/ألم الرأس

Lymphadenopathy/تضخم الغدد اللمفوية

In particular, how would you describe the intensity of the pain symptom?/كيف تقيم شدة الألم؟

no pain (0) لا يوجد ألم

very mild pain (1 - 2) الم طفيف جدا

mild pain (3 - 4)الم طفيف

moderate pain (5 - 6)الم متوسط

severe pain (7 - 8) الم شديد

very severe pain (9 - 10) الم شديد جدا

pain at the injection site(from 0to 10)/ألم مكان الحقنة (من 1 الى 10)

muscle pain/ألم العضلات

headache/ ألم الرأس

In case of temperature> 37 ° C/ في حال ارتفعت حرارتك لأكثر من 37 درجة مأوية:

Yes

No

?/Did you experiance fever once? هل ارتفعت الحرارة مرة واحدة؟

the maximum recorded temperature was> 38 ° C/ هل تجاوزت الحرارة 38 درجة مأوية؟

Have you resorted to antipyretics?/ هل أخذت خافض حرارة؟

For the type of adverse reaction presented .../للمضاعفات التي حصلت:

Yes

No

Have you taken any medications?/ هل أخذت أدوية؟

Did you visit a general practitioner?/ هل زرت الطبيب؟

Have you logged into the emergency room?/ هل زرت غرفة الطوارئ؟

Was hospitalization necessary?/ هل اضطررت لزيارة المستشفى؟

Dopo la sezione 5

Continua alla sezione successiva

Sezione 6 di 11

Dose 2 /الجرعة الثانية

dose 2 of anti-COVID vaccine/الجرعة الثانية للقاح المضاد لفايروس كورونا

Did you take the second dose of anti-covid vaccine?/ هل أخذت الجرعة الثانية لللقاح المضاد لفايروس كورونا؟

*

Dopo la sezione 6

Continua alla sezione successiva

Sezione 7 di 11

side effects

Descrizione (facoltativa)

Did you have any kind of reaction?/ هل تعرضت لمضاعفات؟

Dopo la sezione 7

Continua alla sezione successiva

Sezione 8 di 11

if yes...

Descrizione (facoltativa)

If so, which one?/إذا كان جوابك نعم, ما هي المضاعفات؟

(0-12ساعة)

(12-24ساعة)

Day 1/اليوم 1

Day 2/اليوم 2

Day 3/ اليوم 3

Day 4/ اليوم 4

Day 5/ اليوم 5

Day 6 االيوم 6

Day 7/ اليوم 7

pain at the injection site/ ألم مكان الحقنة

redness at the injection site/ إحمرار مكان الحقنة

swelling at the injection site/ ورم مكان الحقنة

induration at the injection site/ تصلب مكان الحقنة

allergic-urticarial reaction/ تحسس جلدي

anaphylaxis/ حساسية مفرطة

fever (> 37 ° C)/ارتفاع الحرارة أكثر من 37درجة مأوية

diarrhea/ إسهال

vomiting/ تقيئ

asthenia / fatigue/تعب و فقدانالقوة

muscle pain/ألم العضلات

rash/ طفح جلدي

sleepiness / insomnia/ النعاس او الأرق

irritability / nervousness/ التهيج او العصبية

headache/ألم الرأس

Lymphadenopathy/تضخم الغدد اللمفوية

In particular, how would you describe the intensity of the pain symptom?/كيف تقيم شدة الألم؟

no pain (0) لا يوجد ألم

very mild pain (1 - 2) الم طفيف جدا

mild pain (3 - 4)الم طفيف

moderate pain (5 - 6)الم متوسط

severe pain (7 - 8) الم شديد

very severe pain (9 - 10) الم شديد جدا

pain at the injection site(from 0to 10)/ألم مكان الحقنة (من 1 الى 10)

muscle pain/ألم العضلات

headache/ ألم الرأس

In case of temperature> 37 ° C/ في حال ارتفعت حرارتك لأكثر من 37 درجة مأوية:

Yes

No

?/Did you experiance fever once? هل ارتفعت الحرارة مرة واحدة؟

the maximum recorded temperature was> 38 ° C/ هل تجاوزت الحرارة 38 درجة مأوية؟

Have you resorted to antipyretics?/ هل أخذت خافض حرارة؟

For the type of adverse reaction presented .../للمضاعفات التي حصلت:

Yes

No

Have you taken any medications?/ هل أخذت أدوية؟

Did you visit a general practitioner?/ هل زرت الطبيب؟

Have you logged into the emergency room?/ هل زرت غرفة الطوارئ؟

Was hospitalization necessary?/ هل اضطررت لزيارة المستشفى؟

Dopo la sezione 8

Continua alla sezione successiva

Sezione 9 di 11

Anti-body test/فحص المناعة المضادة لفايروس كورونا؟

Descrizione (facoltativa)

Did you perform the anti-SARS-COV2 SPIKE (IgG) antibody assay?/ هل أجريت فحص المناعة المضادة لفايروس كورونا؟

*

Dopo la sezione 9

Continua alla sezione successiva

Sezione 10 di 11

if yes...

Descrizione (facoltativa)

How many days after taking the second dose?كم يوم بعد الجرعة الثانية من اللقاح أجريت الفحص؟

Testo risposta breve

Enter the SARS-COV-2 Spike IgG value/ ما هي النتيجة؟

Testo risposta breve

After receiving vaccination doses, would you repeat the vaccination? بعد تلقي جرعات اللقاح ، هل ستكرر جرعات ايضافيه؟

Dopo la sezione 10

Continua alla sezione successiva

Sezione 11 di 11

comments

Descrizione (facoltativa)

Comments / suggestions/ اقتراحات/ ملاحظات

Testo risposta lunga

**TABLE S2**

Table S2: Prevalence of symptomatic subjects according to the type of vaccine administered

|  | PFZ | AZN | Other | PFZ | AZN | Other |
| --- | --- | --- | --- | --- | --- | --- |
| **1^st^ dose** | ITA (N=1975) | | | LEB (N=822) | | |
| N (%) | 1787 (90.5%) | 154 (7.8%) | 34 (1.7%) | 793 (96.5%) | 12 (1.4%) | 17 (2.1%) |
| Symptomatic N (%) | 1322 (74.0%) | 136 (88.3%)$ | 25 (73.5%) | 297 (37.5%) | 6 (50.0%) | 4 (23.5%) |
| **2^nd^ dose** | ITA (N=1758) | | | LEB (N=722) | | |
| N (%) | 1699/1758 (96.7%) | 36 (2.0%) | 23 (1.3%) | 710 (98.3%) | 2 (0.3%) | 10 (1.4%) |
| Symptomatic N (%) | 1360(80.0%) | 26 (72.2%) | 17 (73.9%) | 425 (59.9%) | 2 (100.0%) | 4 (40.0%) |

PFZ: Pfizer BioNTech, AZN: AstraZeneca;

P<0.01: Chi-square test; $ AZN different from PZF and Other

**TABLE S3**

Table S3: Characteristics of the 2797 participants dividied according to country, vaccine doses and sex distribution after excluding subjects who reported “Injection site pain” as the unique symptom

|  | **All** | **Males** | **Females** |
| --- | --- | --- | --- |
| **ITALIAN COHORT** |  |  |  |
| **1^st^ dose** N. | 1975 | 702 (35.5%)* | 1273 (64.5%)* |
| Age (yrs) | 42.9±16.8^a^ | 45.2±17.0* | 41.7±16.5* |
| Symptomatic N (%) | 632 (32.0%) | 167 (23.8%)* | 465 (36.5%)* |
| N. of symptoms | 3.4±1.8 | 3.0±1.7* | 3.5±1.9* |
| **2^nd^ dose** | 1758/1975 (89.0%) | 631 (35.9%)* | 1127 (64.1%)* |
| Age (yrs) | 43.2±17.1 | 45.6±17.0* | 41.8±17.0* |
| Symptomatic N (%) | 1045 (59.4%) | 298 (47.2%)* | 747 (66.3%)* |
| N. of symptoms | 3.5±2.0 | 3.2±2.0* | 3.8±2.0* |
| **LEBANESE COHORT** |  |  |  |
| **1^st^ dose** N (%) | 822 | 421 (51.2%) | 401 (48.8%) |
| Age (yrs) | 32.5±15.9^a^ | 33.9±16.4* | 31.0±15.2* |
| Symptomatic N (%) | 258 (31.4%) | 108 (25.7%)* | 144 (35.9%)* |
| N of symptoms | 5.6±3.9 | 5.2±3.9 | 5.9±3.9 |
| **2^nd^ dose** | 722/822 (87.8%) | 354 (49.0%) | 368 (51.0%) |
| Age (yrs) | 31.2±14.7 | 32.3±15.1 | 30.2±14.2 |
| Symptomatic N (%) | 403 (55.8%) | 157 (44.4%)* | 246 (66.8%)* |
| N of symptoms | 5.3±3.8 | 4.9±3.8 | 5.6±3.7 |

Data are expressed as numbers (N), mean ± SD, percentages (%).

Asterisks indicate significant differences between sexes (*) p<0.01; symbol (^a^) indicates significant differences between countries after same dose p≤0.01. Statistics by Mann-Whitney test (means) or by chi‐square test (proportions).
